# The Effect of Teff Seed on Hematological Findings and Anthropometric Measurements

**DOI:** 10.4314/ejhs.v32i3.21

**Published:** 2022-05

**Authors:** Eftal Geçgil Demir, Nadide Gizem Tarakçı, Ruken Aslınur Samancı, Merve Cambaz, şeymanur Bilici, Dilara Tuygan, Büşra Çalık, Ayşe Sümeyye Çiftçi

**Affiliations:** 1 Department of Nutrition and Dietetics Institute of Health Sciences, Istanbul Medipol University, 34810, Istanbul, Turkey; 2 Department of Nutrition and Dietetics Faculty of Health Sciences, Istanbul Kent University, 34433, Istanbul, Turkey; 3 Department of Nutrition and Dietetics School of Health Sciences, Istanbul Medipol University, 34083, Istanbul, Turkey

**Keywords:** Teff seeds, Mediterranean diet, obesity, hematological findings, anthropometric measurements

## Abstract

**Background:**

The low incidence of diseases such as celiac, anemia, osteoporosis, and obesity in Ethiopia has aroused interest in the study of teff. The primary objective of this study was to determine the effect of regular consumption of teff seeds on hematological findings and anthropometric measurements in overweight and obese individuals. The secondary objective was to compare these effects of teff seeds with the Mediterranean diet.

**Methods:**

In our study, planned as a cohort study, 28 participants followed the teff seed-containing diet (n=14) and the Mediterranean diet (n=14) for 3 months. To determine nutritional status, a 72-h recall was taken. Anthropometric measurements and hematological findings were recorded at the beginning and end of the study.

**Results:**

There was a significant decrease in fasting blood glucose, cholesterol, LDL, and HDL levels in the teff group (p<0.05). The increase in total protein levels in the teff group was significantly higher than in the Mediterranean diet group (p=0.05). With increased intake of carbohydrates (g) in the teff group, fasting blood glucose levels decreased significantly. There was no significant difference between the two groups regarding anthropometric measurements.

**Conclusion:**

It has been found that the teff seed has no predominance over anthropometric measurements, as compared to the Mediterranean diet, and that it is more effective in improving hematological findings related to obesity. There is a need for more comprehensive studies that also address physical activity, the different types of teff seeds available, and include increased participant numbers.

## Introduction

Teff seed, including white, red, and brown varieties, is of Ethiopian origin and is the smallest among agricultural grains (1,2). The content of teff seed, a gluten-free cereal, includes complex carbohydrates, fiber, unsaturated fatty acids, essential amino acids, polyphenols, and various minerals. Teff seeds have a higher content of fiber, essential amino acids, and minerals (calcium, zinc, phosphate, copper, and iron) compared to cereals such as wheat, rice, sorghum, and corn (2).

Given these contents, it is thought that teff seed may effectively improve obesity. Obesity is a major public health problem associated with increased mortality and morbidity. Obesity increases the risk of arterial hypertension, dyslipidemia, type 2 diabetes, coronary heart disease, cerebral vasculopathy, gallbladder stones, arthropathy, polycystic ovary syndrome, sleep apnea syndrome, and some neoplasms (3).

In Ethiopia, 70% of human nutrition comes from teff seeds. The low incidence of diseases such as celiac, anemia, osteoporosis, and obesity in Ethiopia has generated research interest in teff (4). Although adequate studies have not been done yet, it is believed that teff may benefit health due to its physical and nutritional properties.

The Mediterranean diet is one of the nutritional models applied to prevent obesity and obesity-related diseases. It includes low-glycemic index carbohydrates, high amounts of dietary fiber, antioxidants, polyunsaturated fatty acids, and plant-based proteins and is characterized by intake of w-3 and w-6 fatty acids at a balanced rate. Thanks to these properties, it protects against obesity and obesity (5).

The primary objective of this study was to determine the effect of regular consumption of teff seeds on hematological findings and anthropometric measurements in slightly fat and obese individuals. The secondary objective was to compare these effects of teff seeds with the Mediterranean diet.

## Materials and Methods

**Place of study, time, and sampling selection**: The study was conducted between October and December 2021, with 28 individuals without chronic diseases, aged between 18 and 55 years. The study consisted of female individuals, and the mean age of the participants was 37.0±12.8 years. This study was approved by the Ethics Committee for Non-Interventional Clinical Studies at Istanbul Medipol University (E-10840098-772.02-5236). After the participants were given the necessary information, participants signed a voluntary consent form.

**Data collection and evaluation**: The survey, which questioned the demographic data, was implemented by face-to-face interview. Body Mass Indexes (BMI) of the participants included in the study was in the range of 25–34.9 kg/m^2^.

According to their diet content, the participants were divided into two groups: the teff seed diet (n=14) and the Mediterranean diet group (n=14). For the participants in both groups, isocaloric diets were planned. They were asked to continue their diet programs for 3 months. After each participant's daily energy requirement was specified by the Schofield equation, the diets were calculated to percentages of energy from carbohydrates (50–60%), protein (15–20%), and fat (25–30%). The daily amount of teff seeds (brown) was determined at 20 g (6,7,8) based on similar studies involving gluten-free grains in the past. The amount required during the study was provided by the researcher.

BMI (kg/m^2^) was calculated from weight and height measurements. The researcher measured length, body weight, waist, hips, and upper-middle arm circumference at the beginning and end of the study. The length was measured with Harpenden Stadiometer, without shoes, heels touching each other, feet open 45°, arms flap to side, while the head was in the Frankfort plane. The values were taken in centimeters. Body weights were measured without shoes using a standard weighing tool sensitive to 100 g. The values were taken in kilograms. The waist circumference was measured by the researcher while standing, with the help of an elastic tape in a plane parallel to the floor, while there was a minimum of clothes on the participant.

The 72-h recall was taken at the beginning and end of the study to determine nutritional status. For the food consumption records to be reliable, “Food and Food Photo Catalogue” portions were taught (9). Also, during the study, 72-h recall of all participants was taken every week, and the researcher checked their compliance with the diet.

Biochemical parameters associated with chronic diseases, such as HbA1c, fasting blood sugar, transferrin, albumin, iron, vitamin B12, total protein, vitamin D, total cholesterol, and LDL and HDL cholesterol levels, were analyzed initially and end of the study with the necessary blood samples. The analyzes were carried out in a single center and by the same medical staff. Daily nutrition's energy and nutrients were analyzed using the Nutritional Information System Package Program (BEBİS 8.0) developed for Turkey (10).

**Statistical analysis**: For statistical evaluation of the data obtained, a database was created in IBM SPSS Statistics for Windows (IBM Corp., Version 25.0). The average deviation was presented for descriptive statistics and continuous data, and numeric values and percentage rates were presented for categorical data.

Consistency of continuous data to normal distribution was investigated in statistical comparison of data by Kolmogorov-Smirnov test. Independent two samples t-test for comparing two independent groups in variables that match normal distribution, paired-samples t-test for comparison of dependent variables, and correlation analysis Pearson correlation test was used. Mann Whitney U test for comparison of two independent groups in variables that do not match normal distribution, Wilcoxon Signed Rank Test for comparison of dependent variables, and for correlation analysis, Spearman rank correlation test was performed. Statistical significance was evaluated at a 95% confidence range and p<0.05 level.

## Results

As shown in Table 1, there was no difference in anthropometric measurements at the end of the study between the participants who consumed teff seeds and the participants who consumed the Mediterranean diet (p>0.05). In other words, the changes in anthropometric measurements in both groups were similar.

**Table 1:** Comparison of anthropometric measurements between groups

Variables	Teff (n=14)		Mediterranean Diet (n=14)		Total (n=28)		*p*
		
	x	SD	x	SD	x	SD	
**BMI (kg/m^2^)**	26.7	3.5	27.6	3.7	30.1	3.5	0.463
**Waist Circumference** **(cm)**	93.2	7.9	90.8	22.1	92.1	16.1	0.867
**Hip Circumference** **(cm)**	111.9	8.0	109.2	11.4	110.6	9.7	0.483
**Upper Middle Arm** **Circumference (cm)**	31.7	2.7	33.0	3.9	32.3	3.3	0.347

As shown in Table 2, there was a significant decrease in blood glucose, cholesterol, LDL, and HDL levels in the teff group (p<0.05). Only a significant decrease in cholesterol levels (p<0.05) was observed in the Mediterranean diet group. When both groups were compared, the increase in total protein levels in the teff group was significantly higher than in the Mediterranean diet group (p=0.05).

**Table 2:** Comparison of the hematological findings of the participants at the beginning and end of the study

Biochemical Parameters	Measurement Time	Teff (n=14)			Mediterranean Diet (n=14)			

		x	SD	p_1_	x	SD	p_2_	p_3_
**HbAlc**	Start of Study	5.4	0.3	0.746	5.3	0.5	0.768	0.200
	End of Study	5.3	0.4		5.3	0.5		
	Difference	0.4	2.3		0.5	1.6		
**Hunger blood**	Start of Study	94.3	10.3	0.012*	90.7	9.8	0.432	0.186
**sugar**	End of Study	90.3	7.9		88.9	8.9		
	Difference	4.0	8.0		1.8	8.1		
**Transferrin**	Start of Study	270.4	78.6	0.917	282.5	78.0	0.753	0.937
	End of Study	281.6	62.5		269.7	50.9		
	Difference	23.4	98.0		1.8	21.4		
**Albumin**	Start of Study	4.4	0.5	0.364	4.2	0.7	0.433	0.397
	End of Study	4.3	0.4		4.1	0.5		
	Difference	0.1	0.3		0.1	0.2		
**Iron**	Start of Study	83.7	62.4	0.683	67.5	44.9	0.799	0.512
	End of Study	72.9	36.1		62.2	27.3		
	Difference	13.6	75.5		7.3	39.7		
**Vitamin B12**	Start of Study	243.4	101	0.074	192.3	75.5	0.071	0.495
	End of Study	271.5	127.8		212.6	67.8		
	Difference	30.8	64.3		21.2	40.5		
**Vitamin D**	Start of Study	17.6	9.8	0.480	16.8	10.4	0.084	0.101
	End of Study	19.7	7.8		20.6	6.5		
	Difference	2.6	7.9		4.3	10.3		
**Cholesterol**	Start of Study	199.1	36.6	0.006*	196.3	40.2	0.005*	0.662
	End of Study	186.5	33.6		183.2	38.6		
	Difference	14.5	15.2		13.1	13.9		
**LDL Cholesterol**	Start of Study	122.3	32.0	0.001*	121.8	38.5	0.583	0.116
	End of Study	114.3	27.4		121.4	29.4		
	Difference	8.0	20.2		0.3	21.7		
**HDL Cholesterol**	Start of Study	55.9	11.5	0.009	52.1	12.1	0.746	0.133
	End of Study	53.8	10.5		51.6	10.5		
	Difference	2.0	5.0		0.5	5.6		
**Total Protein**	Start of Study	5.5	3.6	1,000	7.0	0.9	0.028	0.057
	End of Study	7.3	0.1		7.5	0.7		
	Difference	0.5	0.1		0,0	0.5		

p_1_ and p_2_: Paired Samples T Test and Wilcoxon Signed Rank Test; p_3_ : Independent Samples T Test and Mann Whitney U Test

* p<0.05

p_1_: Comparison of the beginning of study and end values of the intervention group

p_2_: Comparison of the starting and end values of the control group

p_3_: Comparison of interventions and control groups between end of study and beginning of study

The relationship between intake of macronutrients and hematological findings of the participants is given in Table 3. With increased intake of carbohydrates (g) in the teff group, hunger blood sugar levels decreased significantly. There was a positive correlation between fat (g) intake and iron mineral levels and a negative correlation with vitamin B12. With increased energy intake in both groups, a decrease in vitamin B12 was observed. In the Mediterranean diet group, we detected a negative correlation between fiber intake and HDL cholesterol levels. Protein (g) intake increased levels of total cholesterol and LDL cholesterol.

**Table 3:** Relationship between intake of macro nutrients and hematologic findings of participants

Nutrient	HbA1C	HBS	Transferrin	Albumin	Iron	Vitamin B12	Total Protein	Vitamin D	Total Cholesterol	LDL	HDL
**Teff**											
**Energy (kcal)**	-0.014	-0.312	-0.084	0.405	0.515	-0.717**	-0.949	0.158	0.80	0.042	0.122
**Carbohydrate (g)**	-0.80	-648.	0.1551	-0.101	0.446	-0.510	0.316	0.028	0.075	0.199	-0.316
**Carbohydrate (%)**	-0.272	0.442	-0.308	-0.388	-0.525	0.369	-0.775	-0.454	-0.473	-0.073	-0.419
**Fiber (g)**	-0.013	-0.277	-0.12,	0.1770	0.351	-0.353	0.348	-0.198	0.284	0.273	0.277
**Protein (g)**	0.175	0.100	-0.244	0.261	-0.003	-0.433	0.738	-0.281	0.262	-0.61	0.021
**Protein (%)**	-0.038	0.447	-0.093	0.079	-0.472	0.418	0.738	-0.234	-0.089	-0.316	0.454
**Fat (g)**	0.096	-0.302	-0.084	0.339	.590*	-.722**	-0.949	0.270	0.403	0.12	-0.050
**Fat (%)**	0.185	-0.396	0.353	0.385	0.521	-0.502	0.316	0.475	0.385	0.201	0.236
**Mediterranean diet**											
**Energy (kcal)**	-0.441	-.588*	-0.21	-0.576	0.293	-647.	0.21	-0.303	0.468	0.340	0.452
**Carbohydrate (g)**	-0.372	-0.535	-0.358	-0.591	0.414	-617.	-0.269	-0.182	0.468	0.459	0.262
**Carbohydrate (%)**	0.255	-0.068	-0.374	0.103	0.396	0.106	-0.710	0.279	-0.071	0.307	-0.326
**Fiber (g)**	-0.008	-0.15	-0.307	0.042	0.623*	0.031	-0.689	-0.237	-0.061	-0.076	-0.746
**Protein (g)**	-0.555	-0.268	-0.184	-0.317	0.451	-0.326	0.092	-0.219	.718**	.595*	0.145
**Protein (%)**	0.177	0.244	-0.289	0.327	0.177	0.427	0.000	.591*	0.241	0.441	-0.102
**Fat (g)**	0.017	-0.537	0.167	-.700.	-0.217	-0.673	0.200	-0.322	0.338	-0.099	0.259
**Fat (%)**	-0.026	-0.60	0.287	-0.242	-0.405	-0.321	0.429	-0.204	-0.138	-0.420	0.424

Pearson correlation and Spearman rank correlation were used.

* p<0.05

** p<0.01

The relationship between the participants' macronutrient intake and anthropometric measurements is shown in Table 4. The upper-middle arm circumference increased as energy intake increased and fat (g) intake increased in the teff group. In addition, an increase in fat (g) was detected around the waist.

**Table 4:** Relationship between respondents' macro nutrient intake anthropometric measurements

Nutrient	BMI	Waist Circumference	Hip Circumference	Upper Middle Arm Circumference
**Teff (n=14)**				
**Energy (kcal)**	0.438	0.63	0.184	.623*
**Carbohydrate (g)**	0.262	0.075	-0.001	0.482
**Carbohydrate (%)**	-0.268	-0.151	0.131	-0.210
**Fiber (g)**	0.094	0.322	0.405	0.303
**Protein (g)**	0.108	0.362	0.505	0.263
**Protein (%)**	-0.238	-0.272	0.051	-0.399
**Fat (g)**	0.489	.564*	0.388	.694**
**Fat (%)**	0.374	0.259	-0.082	0.412
**Mediterranean diet (n=14)**				
**Energy (kcal)**	0.324	0.24	0.423	0.418
**Carbohydrate (g)**	0.483	0.204	0.444	0.480
**Carbohydrate (%)**	0.195	-0.028	-0.010	0.025
**Fiber (g)**	0.199	-0.017	0.344	0.296
**Protein (g)**	0.350	0.452	0.347	0.406
**Protein (%)**	0.081	0.174	-0.306	0.051
**Fat (g)**	-0.047	-0.044	0.207	0.196
**Fat (%)**	-0.186	-0.14	0.069	-0.002

Pearson correlation and Spearman rank correlation were used.

* p<0.05

** p<0.01

## Discussion

The incidence and prevalence of obesity increase daily, causing many chronic diseases and negatively affecting people's lives (11). It is believed that teff seed, which is the basis of human nutrition in Ethiopia, can be useful in treating obesity because of its rich micronutrient content. Accordingly, the effect of teff seed on hematological findings and anthropometric measurements in slightly fat and obese individuals is important for public health.

Known as a high-energy, nutritious food that helps treat diabetes and cardiovascular diseases, teff has slowly digestible starch and complex carbohydrates content. Complex carbohydrates account for 80%, and starch accounts for about 73% of teff grain (12). Teff seeds have a lower glycemic index and higher fiber content than cereals (13). Its amylose content can explain its low glycemic index properties, amylose-lipid complexes that prevent starch digestibility, low starch damage, and high gelatinization temperature lowering the sensitivity to the α-amylase (14). It is argued that the glycemic index reflects the postprandial glucose response of carbohydrate-containing foods, and its effect is associated with the fiber content of consumed foods. High fiber content can be explained by the fact that whole grains contain more fiber than peeled, and small grains contain bran, which is rich in fiber (15). In a study conducted by DeBoer et al. (2018), teff was found to have low glucose and insulin response (16).

Similarly, in this study, there is no significant change in blood glucose levels in the Mediterranean diet group compared with the beginning of the study, while in the teff group, there is a significant reduction in blood glucose levels (p<0.05). Also, a decrease in hunger blood sugar was observed in the teff group when carbohydrates (g) increased. This effect of teff is a privilege compared to other gluten-free cereals, especially in patients with diabetes and celiac progressing together.

As a result of this study, it was determined that there was no significant change in total and LDL cholesterol levels in the Mediterranean diet group and significantly decreased in the teff group (p<0.05). In addition, there was an increase in total and LDL cholesterol levels when protein (g) increased in the Mediterranean diet group. A similar result was not encountered in the group that consumed teff seeds. Given the daily consumption of cereals, they can be a good source of essential fatty acids (2). The predominant fatty acids in teff seed are oleic acid (32.41%) and linoleic acid (23.83%), respectively. The ratio of LA:ALA is 7:1, and the content of unsaturated fatty acids is higher than that of other cereals (2,17,18). The fatty acid content of teff seed plays a critical role in the nutritional value, reducing the risk of developing cardiovascular disease, inflammation, cancer, and other diseases (18). It is also reported that due to its rich composition in total and soluble fiber, it can lower blood cholesterol (2). This study shows that teff seed plays an important role in regulating blood cholesterol levels and other existing studies.

In this study, a decrease in the level of iron minerals in the blood and an increase in transferrin level was found when the teff group was examined (p>0.05). On the contrary, another study conducted by Alaunyte et al. (2014) showed a decrease in transferrin level (19). Teff seeds contain higher iron minerals than other cereals (20). Since it is a naturally rich grain in iron, it is considered a more efficient and reliable strategy for treating iron deficiency (21). It is noted that the prevalence of anemia in Ethiopia is lower than in other regions (20,22). Despite this feature, it is believed that the cause of this decrease may be a decrease in the bioavailability of iron minerals due to the presence of phytic acid and inositol phosphates (23). A study conducted by Delil et al. (2018) in Ethiopia found that 99.7% of pregnant women consumed teff, and 75.8% did not experience anemia (24). In another study, consumed by 77% of female individuals, teff ensured that sufficient iron minerals were obtained. It was also observed that the risk of intestinal parasites and associated anemia was significantly reduced in proportion to handwashing habits (25). Similarly, in a 6-week dietary intervention in female athletes in developing countries, anemia was not observed only in the Ethiopian athletes, and this was suggested to be because of teff consumption (19).

Gluten-free products, often used by celiac patients, may be insufficient to meet daily vitamin and mineral requirements, and therefore iron supplement is taken. Food supplements can often cause side effects such as constipation, nausea, and diarrhea while increasing the amount of iron taken in the diet. Unlike other cereals, teff is rich in iron content, and there is no need for reinforcement (22). Teff's LA:ALA ratio is ideal, and n-3 fatty acids are reliable and effective in treating anemia (26). In this study, iron values that increase when fat (g) is increased in the teff group suggest that teff can be used as a natural source of nutrients in the treatment of anemia in celiac patients.

The content of both the teff and the Mediterranean diet group is plant-based protein. The Mediterranean diet is rich in unsaturated fats and plant-based proteins, limited in animal-based protein (27). In this study, in both groups, energy and fat (g) increase and decreases in B12 values were associated with limiting dietary content from animal-based proteins.

When the teff and Mediterranean diet groups were compared, we found that the change in total protein levels that provide information about individuals' overall health and nutritional status was greater in the teff group. In connection with this, it can be said that teff consumption contributes to raising total protein levels to the reference range. Teff seed has an excellent amino acid composition and, consequently, high protein content, as it contains all 8 essential amino acids for humans (28). Based on our findings, the positive effect of nutrition on serum albumin and globulin ratio can be expressed thanks to protein content in the teff group. Albumin and globulin are two markers that reflect albumin/globulin ratio inflammation (29). Concerning this, it can be said that the participants in the teff group have a low risk of inflammation.

While there was no significant difference between the teff and the Mediterranean diet group regarding weight loss, there was a significant decrease in BMI values in both groups (p=0.00). This might be associated with the energy constraints made. There was no positive effect of teff on anthropometric measurements (p>0.05).

This study has some limitations. First, healthy eating, regular exercise, weight control, and quitting smoking have increased HDL cholesterol levels (30). Inquiry of the state of physical activity of participants in this study is a limitation. As fiber consumption increases in the Mediterranean diet group, the value of HDL cholesterol decreases, independent of nutrition; this may be associated with a lack of physical activity.

Second, 28 participants were included in our study. It is recommended that the effects of teff seed on health be supported by studies involving more participants. Finally, this study used only one kind of teff seed. Comparisons with other varieties have not been made due to the major consumption of brown teff seed in Turkey. In other studies, in this field, the effects on health can be compared using different teff seed types.

As a result, it has been found that the Teff seed has no predominance over anthropometric measurements, as compared to the Mediterranean diet, and that it was more effective in improving hematological manifestations related to obesity. The effects of regular teff consumption increased total protein levels reducing blood sugar total and LDL cholesterol. There was no effect other than energy constraint when examining its relationship with anthropometric measurements. Teff based diets are recognized worldwide, and the interest of consumers that pay attention to its health benefits increases daily, and hence other studies are required to be carried out in this field to obtain empirical data on the effects of teff seed on anthropometric measurements, biochemical findings, and health.
